# PI3Kɑ inhibition reduces obesity in mice

**DOI:** 10.18632/aging.101075

**Published:** 2016-11-04

**Authors:** Elena Lopez-Guadamillas, Maribel Muñoz-Martin, Sonia Martinez, Joaquin Pastor, Pablo J. Fernandez-Marcos, Manuel Serrano

**Affiliations:** ^1^ Tumor Suppression Group, Spanish National Cancer Research Center (CNIO), Madrid E28029, Spain; ^2^ Experimental Therapeutics Programme, Spanish National Cancer Research Center (CNIO), Madrid E28029, Spain; ^3^ Bioactive Products and Metabolic Syndrome Group, Madrid Institute of Advanced Studies (IMDEA) in Food, CEI UAM+CSIC, Madrid E28049, Spain

**Keywords:** PI3K, obesity, energy expenditure, diabetes, metabolism

## Abstract

Partial inhibition of PI3K is one of the best-validated and evolutionary conserved manipulations to extend longevity. The best known health beneficial effects of reduced PI3K are related to metabolism and include increased energy expenditure, reduced nutrient storage, and protection from obesity. We have previously shown that a dual chemical inhibitor of the alpha and delta PI3K isoforms (CNIO-PI3Ki) reduces obesity in mice and monkeys, without evident toxic effects after long-term treatment. Here, we dissect the role of the alpha and delta PI3K isoforms by making use of selective inhibitors against PI3Kɑ (BYL-719 also known as alpelisib) or PI3Kδ (GS-9820 also known as acalisib). Treatment of mice with the above mentioned inhibitors indicated that BYL-719 increases energy expenditure in normal mice and efficiently reduces body weight in obese (ob/ob) mice, whereas these effects were not observed with GS-9820. Of note, the dose of BYL-719 required to reduce obesity was 10-times higher than the equivalent dose of CNIO-PI3Ki, which could suggest that simultaneous inhibition of PI3K alpha and delta is more beneficial than single inhibition of the alpha isoform. In summary, we conclude that inhibition of PI3Kɑ is sufficient to increase energy expenditure and reduce obesity, and suggest that concomitant PI3Kδ inhibition could play an auxiliary role.

## INTRODUCTION

The first gene mutation found to extend longevity in an animal was in the age-1 gene of *Caenorhabditis elegans* [1], which was later shown to encode the catalytic p110alpha subunit of class I phosphatidylinositol-4,5-bisphosphate 3-kinase (PI3Kɑ) [2]. PI3Kɑ mediates the signaling of numerous factors, being insulin and insulin-like growth factor 1 (IGF1) of special relevance. Indeed, partial genetic reduction of the insulin and IGF1 signaling (IIS) pathways at different levels extends longevity in worms, flies and mice [3]. For example, similar to worms, heterozygous inactivation of the gene encoding PI3Kɑ also extends longevity in mice [4]. Despite the strong link between PI3K down-modulation and longevity, it remains unclear which of its multiple physiological consequences are responsible for the beneficial effects on health and aging. A main function of the PI3K pathway is to activate anabolism and nutrient storage and, conversely, a consistent observation in a variety of genetic mouse models with partial PI3K down-modulation is their higher energy expenditure and protection from obesity [5]. Therefore, the beneficial metabolic effects of reduced PI3K signaling could explain, at least in part, the improved healthspan and delayed aging. Furthermore, inhibition of the PI3K downstream effector mTOR by rapamycin also increases longevity [6] and reduces body weight [7].

The above-described genetic evidences make very attractive the possibility that moderate inhibition of PI3K with small chemical compounds could have beneficial health effects. Indeed, two selective inhibitors of PI3Kɑ, PIK75 and A66, reduce body weight in normal lean mice but present negative effects including reduced locomotor activity [8]. On the other hand, we have shown that a chemical PI3K inhibitor with good oral bioavailability and pharmacokinetics, named CNIO-PI3Ki, can efficiently reduce adiposity in obese mice and in obese Rhesus monkeys in the absence of detectable toxicities [9]. Of note, CNIO-PI3Ki not only inhibits PI3Kɑ but also PI3Kδ [9]. PI3Kδ is one of the four class I PI3K isoforms [10] and is mainly involved in the regulation of immune cells [11]. Interestingly, there is a strong association between inflammation of the adipose tissue and the pathological manifestations of obesity [12]. Based on this, it is conceivable that the inhibition of PI3Kδ could also contribute to the beneficial metabolic effects of CNIO-PI3Ki.

In this report, we use selective inhibitors of PI3Kɑ and PI3Kδ in mice to determine their efficacy in reducing obesity and elevating energy expenditure.

## RESULTS

### Differential effects of PI3K inhibitors on obesity in ob/ob mice

**Figure 1:**
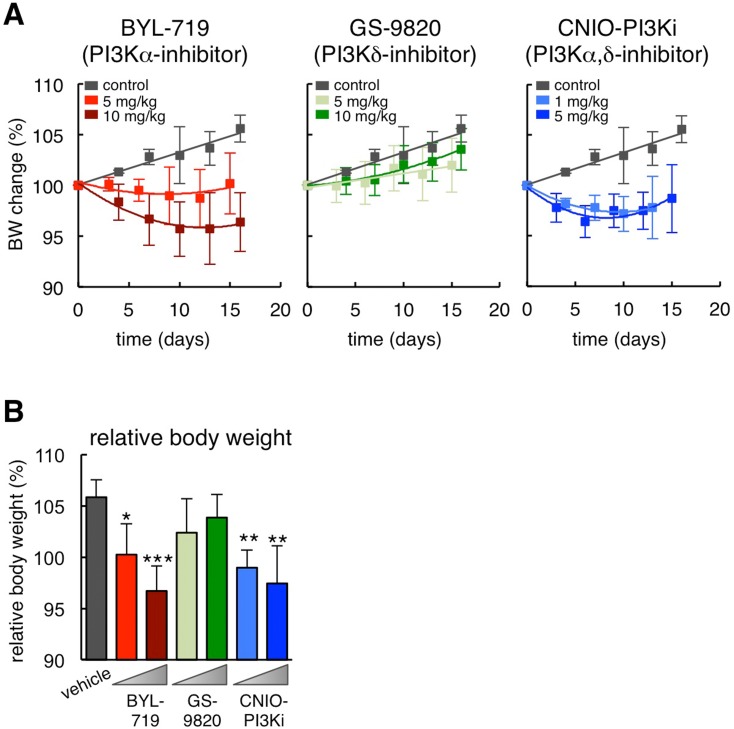
Differential effects of PI3K inhibitors on obesity in ob/ob mice (**A**) Body weight change relative to day 0 during daily dosing of the indicated PI3K inhibitors (n=10 per group, ob/ob males, 20 weeks old). The vehicle treated group is the same for the three graphs. (**B**) Relative body weight change at the end of the treatment (day 15 or 16) of the same experimental groups shown in panel A. Values correspond to average ± s.d. Statistical significance was determined by the two-tailed Student's t-test relative to vehicle controls: *p < 0.05, ** *p* <0.01, *** *p* <0.001.

To dissect the relative contribution of PI3Kɑ and PI3Kδ inhibition in the reduction of obesity, we treated obese hyperphagic ob/ob mice with a selective PI3Kɑ inhibitor, BYL-719 [13], or with a selective PI3Kδ inhibitor, GS-9820 (also known as CAL-120) [14]. Remarkably, BYL-719 reduced body weight after 15 days of treatment to a similar extent as CNIO-PI3Ki, whereas GS-9820 had no significant effect at the same doses as BYL-719 (Figure 1A and 1B). It should be noted that 10 mg/kg of GS-9820 is sufficient to reduce the growth of multiple myeloma xenografts in mice [15]. Interestingly, CNIO-PI3Ki at 1 mg/kg was as effective as BYL-719 at 10 mg/kg. The higher efficien-cy of CNIO-PI3Ki may be due to a number of reasons, such as for example a better pharmacokinetics, but it could also reflect a contribution of PI3Kδ inhibition in the reduction of obesity in the context of simultaneous PI3Kɑ inhibition. We conclude that inhibition of PI3Kɑ is sufficient to reduce obesity, but we cannot exclude an additional auxiliary benefit due to the concomitant inhibition of PI3Kδ.

### On-target effects of PI3Kɑ inhibition in ob/ob mice

**Figure 2:**
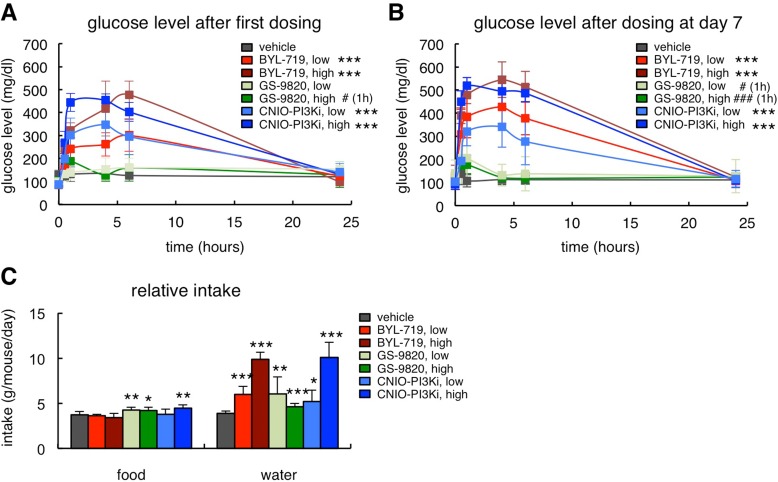
On-target effects of PI3Kɑ inhibition in ob/ob mice (**A**) Glucose serum excursions after a single administration of vehicle, BYL-719 (5 or 10 mg/kg), GS-9820 (5 or 10 mg/kg) or CNIO-PI3Ki (1 or 5 mg/kg) by oral gavage (n=10 per group, ob/ob males, 20 weeks old). Treatments and measurements were done under ad libitum feeding. (**B**) Glucose serum excursions measured 7 days after the beginning of the treatment in the same mice as in Figure 2A. (**C**) Relative food and water intake in the same mice as in Figure 1A. The results correspond to the average daily food and water intake from days 7 to 12. Values correspond to average ± s.d. Statistical significance was determined by the two-tailed Student's t-test relative to vehicle controls: * *p* < 0.05, ** *p* <0.01, *** *p* <0.001. In Figures 2A and 2B, significant differences were found from 0.5 h to 6 h post-gavage, except for acalisib that is significant only at 1 h, indicated with # (1h).

PI3Kɑ is involved in the signaling of insulin and, therefore, hyperglycemia is an expected on-target effect of PI3Kɑ inhibitors. In this regard, we have previously reported that, in lean mice, CNIO-PI3Ki at 15 mg/kg produces a moderate glycemic excursion, within physiological range (up to 150 mg/dl of serum glucose), and reversible within 8 h [9]. Obese ob/ob mice are insulin resistant and therefore their glycemic excursions were severe (up to 500 mg/dl) in the case of the two PI3Kɑ inhibitors, CNIO-PI3Ki and BYL-719, whereas GS-9820 had a comparatively minor effect (Figure 2A). It is important to note that the hyperglycemia produced by 1 mg/kg CNIO-PI3Ki was less severe than the one produced by 10 mg/kg BYL-719 (Figure 2A), being both treatments equally efficient in reducing obesity (Figures 1A and 1B). The hyperglycemic peaks were in all cases fully normalized after 24 h (Figure 2A). Furthermore, in mice that had been treated daily with the PI3K inhibitors for 7 days, glucose levels were also normal 24 h after the last administration of inhibitors (Figure 2B). The severe hyperglycemia produced in ob/ob mice was also reflected by their increased water intake, a compensatory response to reduce hyperglycemia (Figure 2C). Finally, we observed a modest increase in food intake in mice treated with GS-9820 or with high-dose CNIO-PI3Ki (Figure 2C).

**Figure 3:**
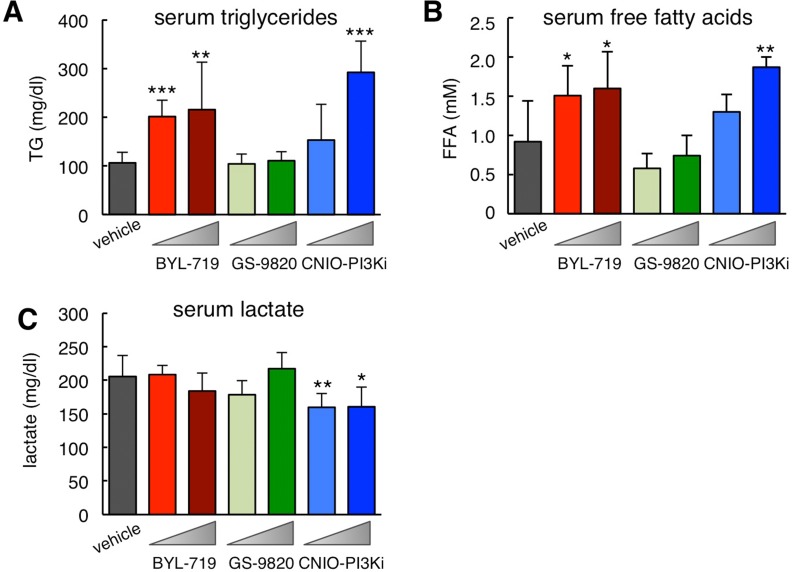
Biochemical serum profile of ob/ob mice after treatment with PI3K inhibitors (**A**) Serum triglycerides (TG) in the same mice as in Figure 1A measured 3-4 hours after the last dosing at the end of the treatment (always under ad libitum feeding). (**B**) Serum free fatty acids (FFA) as in panel A. (**C**) Serum lactate as in panel A. Values correspond to average ± s.d. Statistical significance was determined by the two-tailed Student's t-test relative to vehicle controls: * *p*< 0.05, ** *p* <0.01, *** *p* <0.001.

Inhibition of insulin signaling not only affects glucose homeostasis but also lipid metabolism. Therefore, another anticipated on-target effect of PI3Kɑ inhibition in the white adipose tissue is a reduction in the uptake of dietary triglycerides (TG) and an increase in lipolysis that results in elevated serum free fatty acids (FFA) [16,17]. These effects are recapitulated by treatment with rapamycin, which inhibits mTOR, a key down-stream effector of PI3K [18]. In agreement with this, mice treated for 15 days with BYL-719 or CNIO-PI3Ki presented increased serum TG (Figure 3A) and FFA (Figure 3B), while GS-9820 had no detectable effect on serum lipids.

Partial down-modulation of PI3K has been reported to reduce serum lactate levels due to increased mitochondrial oxidative phosphorylation [19] and this effect has also been observed upon treatment with rapamycin [20]. Interestingly, mice treated for 15 days with 1 mg/kg CNIO-PI3Ki had a significant reduction in serum lactate, whereas this was not observed with BYL-719 or with GS-9820 at 10 mg/kg (Figure 3C). All together, we validate that BYL-719 and CNIO-PI3Ki are inhibiting PI3Kɑ *in vivo* by using a number of on-target readouts (hyperglycemia, increased serum lipids, and reduced serum lactate), whereas these effects are absent upon treatment with GS-9820.

### Differential effect of PI3K inhibitors in energy expenditure

**Figure 4:**
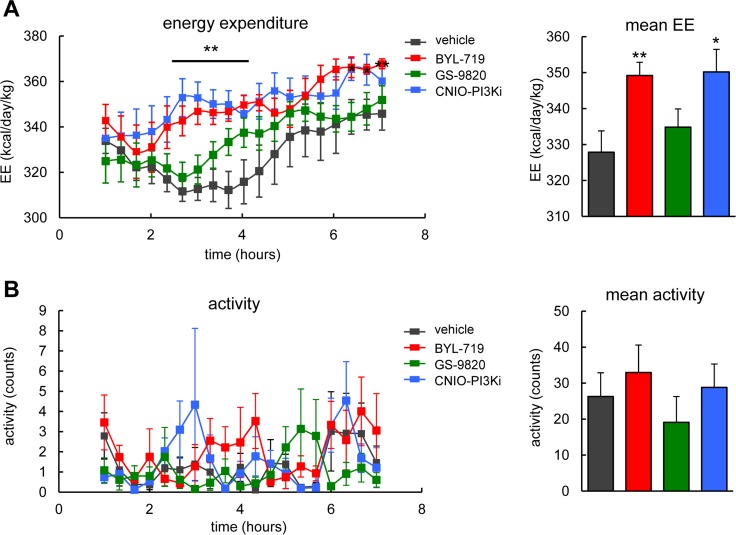
Energy expenditure and activity of wild-type mice after treatment with PI3K inhibitors (**A**) *Left*, energy expenditure (EE). *Right*, mean EE. Calorimetry of male WT mice, under ad libitum feeding, after a single oral dose of 15 mg/kg of the indicated PI3K inhibitors (n=7-8 per group, C57BL6 males, 20 weeks old). The line and asterisks indicate that CNIO-PI3Ki and BYL-719 are significantly different relative to vehicle at these time points. (**B**) *Left*, activity. *Right*, mean activity. Activity was measured in the same mice as in panel A. Values correspond to average ± s.e.m. Statistical significance was determined by the two-tailed Student's t-test relative to vehicle controls: * *p* < 0.05, ** *p* <0.01.

The PI3K pathway is a major inducer of anabolism and, therefore, genetic down-modulation of PI3K by PTEN or treatment of mice with CNIO-PI3Ki elevates energy expenditure [19,21]. We used lean wild-type mice to test the effect of a single dose (15 mg/kg) of each of the three PI3K inhibitors on energy expenditure. Interestingly, BYL-719, but not GS-9820, elevated energy expenditure during 7 h after oral administration in a similar way as CNIO-PI3Ki (Figure 4A). No changes in locomotor activity were observed in any of the treated mice (Figure 4B). Normal locomotor activity suggests the absence of severe toxic effects, and also excludes the possibility that increased physical activity could be the underlying reason for the increased energy expenditure. We conclude that PI3Kɑ, but not PI3Kδ, regulates energy expenditure in mice.

## DISCUSSION

Previous work by us has shown that a dual PI3Kɑ and PI3Kδ inhibitor, namely CNIO-PI3Ki, reduces obesity and elevates energy expenditure in mice. Here, we have used selective inhibitors of PI3Kɑ and PI3Kδ and we have concluded that PI3Kɑ is the main isoform responsible for these beneficial metabolic effects. These observations are in agreement with the reported protection against aging-associated obesity of mice with reduced expression of PI3Kɑ [4]. It should be noted that CNIO-PI3Ki seems more efficient that BYL-719 in reducing obesity. Although the differential efficacy of the two inhibitors can have several explanations, one possibility is that PI3Kδ inhibition, although not sufficient by itself, contributes to reduce obesity in the context of simultaneous PI3Kɑ inhibition.

In addition, we have validated some on-target effects of PI3Kɑ inhibition produced as a consequence of reduced insulin signaling, including elevated levels of serum glucose, and serum lipids. In contrast, PI3Kδ inhibition by GS-9820 did not produce any of these effects even administered at a dose that was shown to have anti-tumoral effectiveness [15], thereby confirming its minor role in insulin signaling. The most dramatic on-target effect of PI3Kɑ inhibition was hyperglycemia, which in the case of the ob/ob mice was severe due to their diabetic condition. However, it is important to note that in normal lean mice, the glycemia induced by PI3Kɑ inhibition is within physiological range [9]. In summary, we conclude that moderate pharmacological inhibition of PI3Kɑ is sufficient to elicit the metabolic beneficial effects of reduced PI3K signaling. Our results, however, leave open the possibility that inhibition of PI3Kδ could also contribute to these effects in the context of concomitant PI3Kɑ inhibition.

## MATERIALS AND METHODS

### Ethics statement

All animal procedures were approved by the CNIO-ISCIII Ethics Committee for Research and Animal Welfare (CEIyBA) and the Community of Madrid, and conducted in accordance to the recommendations of the Federation of European Laboratory Animal Science Associations (FELASA) and the institutional guidelines.

### Mouse experimentation

Mice were housed under specific pathogen free (SPF) conditions, at 22°C, and with 12 hours dark/light cycles (light cycle from 8 am to 8 pm). Mice were fed with standard chow diet (Harlan Teklad 2018, 18% of fat-based caloric content). Ob/ob C57BL6J mice were purchased from Charles River Laboratories. Wild-type C57BL6J/Ola.Hsd mice were purchased from Harlan. All mice used were males of 20 weeks of age. PI3K inhibitors were administered daily by oral gavage during 15 or 16 days as follows, BYL-719 (5 and 10 mg/kg) and GS-9820 (5 and 10 mg/kg), CNIO-PI3Ki (1 and 5 mg/kg), dissolved in PEG-300 (Sigma) and 10% N-methyl-2-pyrrolidone (Sigma).

### Serum analyses

For glucose excursions (Figure 2A and B), mice under ad libitum feeding were treated with a single dose of the indicated PI3K inhibitors by oral gavage (at 10:00 am) and blood was collected from the tail tip for the determination of glucose (Glucocard strips; A. Meranini). For all the other serum analyses, mice at the end of their corresponding daily treatments for 15-16 days received a final dose of treatment and were sacrificed 3-4 h later (always under ad libitum feeding). Blood was collected from post-mortem heart puncture. Serum free fatty acids were quantified by a colorimetric assay (BioVision #K612-100). Triglycerides and lactate levels were measured using the ABX PENTRA 400 clinical chemical analyzer (Horiba ABX Diagnostics).

### Indirect calorimetry and activity

Indirect calorimetry was performed following standard methods using Oxylet System metabolic chambers (Panlab Harvard Apparatus). Acclimatization of mice to the measurement cages was three days prior to data recording. Mice under ad libitum feeding were treated with a single dose of 15 mg/kg for each PI3K inhibitor (BYL-719, GS-9820 and CNIO-PI3Ki) by gavage. The volumes of consumed O_2_ (VO_2_) and eliminated CO_2_ (VCO_2_) were recorded every 24 min (8 simultane-ous metabolic chambers) for the following 7 hrs. Room temperature was constantly kept at 21°C. Energy Expenditure (EE) was calculated as EE=3.815+(1.232x (VCO_2_/VO_2_)) x VO_2_ x 1.44. Mouse activity was recorded in time intervals of 20 min during the whole measurement period.

### Statistical analyses

Values are expressed as mean ± s.d. (Figures 1 to 3) or mean ± s.e.m (Figure 4). Statistical analyses were performed using unpaired two-tailed Student's t-test and differences with *P* values of <0.05 were considered to be statistically significant (* *p*<0.05, ** *p*<0.01, *** *p*<0.001). Statistical analyses were performed using Excel or GraphPad Prism software.
